# Re‐theorizing the Sexual Minority Closet: Evidence From Queer South Asian Women

**DOI:** 10.1111/cars.70016

**Published:** 2025-09-17

**Authors:** Sonali Patel

**Affiliations:** ^1^ Department of Sociology University of British Columbia Vancouver Canada

**Keywords:** identity concealment, intersectionality, LGBTQ, post‐closeted, queer women of color

## Abstract

Scholarship generally assumes the closet is a place of safety from the perceived risks associated with coming out. However, this overlooks its function as a source of violence, particularly for those belonging to multiple marginalized communities. Drawing on 40 qualitative interviews with second‐ and 1.5‐generation queer South Asian women (QSAW) in Canada, this article offers a re‐theorization of the closet as a dual site of safety and violence. My findings show that the convergence of sexual expectations of coming out with ethnic demands of staying closeted exacerbates QSAW's vulnerability to violence from family, the LGBTQ+ community, and intimate partners, who use violence as a tool to enforce, contest, and exploit the closet, respectively. Ultimately, the results stress the dangers of pressuring QSAW to come out to their parents. The findings are significant for understanding the intersectional complexities of the closet.

## FRENCH ABSTRACT

De manière générale, la littérature scientifique conçoit le placard comme un espace sécuritaire face aux risques perçus reliés au *coming out*. Toutefois, cette perspective néglige la fonction du placard comme une source de violence surtout pour les individus appartenant à plusieurs communautés marginalisées. En se basant sur quarante entretiens qualitatifs menés au Canada auprès de femmes sud‐asiatiques queer (QSAW) de seconde et 1.5‐génération, cet article propose une re‐théorisation du placard comme un lieu double où l'on retrouve la sécurité et la violence. Mes résultats révèlent que la convergence entre les attentes sexuelles reliées au *coming out *et les exigences ethniques de rester dans le placard accroît la vulnérabilité des QSAW à la violence provenant de la famille, de la communauté LGBTQ+, et des partenaires intimes. Cette violence est utilisée comme un outil pour, respectivement, renforcer, contester ou exploiter le placard. Ultimement, les résultats mettent en lumière les risques inhérents à la pression exercée sur les QSAW pour qu'elles fassent leur *coming out* auprès de leurs parents. Ces résultats sont essentiels pour comprendre la complexité intersectionnelle du placard.

## 1 Introduction

Since the emergence of the gay and lesbian^1^ liberation movement in the 1960s, the Western LGBTQ+ community has encouraged its members to embrace public queer^2^ lives (Seidman 2002). Over the subsequent decades, expanding state support (Smith 2011) and improving societal acceptance of sexual minorities (Andersen and Fetner 2008; Keleher and Smith 2012) made it viable to “live beyond the closet” (Seidman 2002, 63). Yet, doing so can pose challenges for South Asian sexual minorities, who face incongruent cultural constraints that necessitate concealment (Bacchus 2017). When ethnic communities prescriptively enforce heterosexuality (Jaspal 2012), stigmatize and repress homosexuality (Adur 2018), and value preserving familial honor (Siraj 2018), coming out may be untenable (Jaspal and Siraj 2011), rendering the closet a necessary refuge (Patel 2024). However, the extent to which it offers protection—given Western coming‐out norms—remains unclear. Existing scholarship generally assumes the closet is a place of safety. That is, people hide their sexuality due to the perceived risks of heterosexual hostility associated with coming out (Bry et al. 2017; Feinstein et al. 2020; Jackson and Mohr 2016; Pachankis 2007). This literature privileges monolithic analyses that disregard the influence of ethno‐cultural factors and, consequently, overlooks the possibility that the closet can also be a site of violence. When scholars do address animosity toward closeted individuals, they overwhelmingly center on outing or the threat of exposure during the closeted era, positioning harm as contingent upon leaving the closet. This elides the manifestation of violence inside the closet, independent of outness, and implicitly dismisses the potential for any such harm during the post‐closeted era, thereby reinforcing the presumption that the closet itself is a place of safety. To address this shortfall, this article aims to understand the intersectional complexities of navigating the closet. I focus on queer South Asian women (QSAW) because their experiences at the axis of Western and non‐Western cultures, alongside homophobia, racism, and sexism, provide valuable insight for understanding the intersectional implications of the closet.

This article qualitatively explores how QSAW in Canada experience the closet, particularly during the post‐closeted era. By considering the convergence of ethnic expectations to stay closeted (Bacchus 2017; Patel 2024; Siraj 2018) with sexual pressures to come out (Patel 2019, 2021) among 40 interviewees, I propose a re‐theorization of the closet as a dual site of safety and violence. I argue that being closeted increases QSAW's vulnerability to violence from family, LGBTQ+ community members, and intimate partners. While expectations to remain closeted affect South Asians of all genders (Jaspal 2012; Siraj 2018), the responsibility of preserving familial honor disproportionately burdens women (Gilbert et al. 2004). This exacerbates closet‐related complexities, making it harder for QSAW to come out in ethnic contexts (Bacchus 2017). To avoid glossing over these entwined patriarchal and cultural consequences, I focus on QSAW.

Being in the closet means concealment is enforced in at least one domain of a person's life, even if their identity is known to LGBTQ+ peers, intimate partners (Ponse 1976), friends (Kilday and Nash 2017), or parents (Patel 2024). To best capture intersectional experiences, I draw on my concept of the “culturally expansive closet,” which I theorized in a previous article using the same dataset (see Patel 2024, 10). This framework broadens the closet's boundaries to include those who comply with parental demands to hide their sexuality from extended family. In this view, revealing one's identity to parents does not constitute being out; rather, outness is demarcated by disclosure to one's ethnic community. This article does not propose that all QSAW are closeted, nor does it suggest that all South Asian parents are violent towards their children; rather, it reflects the respondents’ realities.

I define violence as a relational strategy used to control or constrain an individual's choices and autonomy (Schäfer 2024). The type of violence enacted may vary by relationship. Lateral violence in LGBTQ+ communities often manifests as microaggressions: brief verbal, behavioral, or attitudinal indignities (Nadal 2013) that, whether intentional or not, serve to invalidate and delegitimize the intersectional realities of marginalized LGBTQ+ individuals (Vaccaro and Koob 2019). Violence within intimate partnerships or parent‐adult child ties may encompass a range of behaviors, including any physical, sexual, emotional, and financial abuse, as well as neglect (Dutton 2006; Gracia et al. 2020; Lawson 2012; Malley‐Morrison and Hines 2012).

The findings advance extant scholarship in two ways. First, they add nuance to existing conceptualizations of the closet as a place of refuge by also establishing it as a site of violence. Second, they contribute to an emerging body of literature on diasporic QSAW's experiences, which enables a greater understanding of the issues endured by this historically underrepresented group.

## 2 Understanding the Closet

The closet is often conceived in relation to coming out. Whereas coming out entails openly disclosing one's LGBTQ+ identity (Ghaziani and Holmes 2023; Mohler 2000), the closet refers to hiding one's sexuality (Seidman 2002; Siraj 2018). It is a relational construct that is maintained through social ties (Adams 2010; Doyle and Barreto 2023).

The closet is not a simple binary in which one is either out or in (Sedgwick 2008); rather, there are different degrees of closetedness, ranging from full to partial. One is fully closeted when only the self or both parties of a queer dyad are aware of one's homosexuality (Ponse 1976, 316). Partially closeted are those who hide their sexuality in some social relationships and contexts while disclosing it in others (Doyle and Barreto 2023; Schmitz and Tyler 2018). Being closeted traditionally meant “being secretive about the gay self” with heterosexuals (Ponse 1976, 316) and especially with one's parents (D'augelli et al. 2008; D'Augelli and Hershberger 1993). Recent scholarship expands the definition of the closet to include those who resume concealment after coming out in a relational context. For instance, if parents react negatively to initial disclosure, then the child may continue hiding meaningful information about their sexuality, such as romantic relationships (Meidlinger and Hope 2014). Likewise, if parents deny the identity disclosure and demand it be hidden from extended family, then the compliant child is still considered closeted. The closet is thus a non‐linear trajectory (Patel 2024).

The closet and concealment are related but distinct: all closeted individuals conceal, but not everyone who conceals is closeted. Concealment is an interactional strategy for managing stigmatized identities (Goffman 1963), involving varying degrees of non‐disclosure (Doyle and Barreto 2023). The closet's definition, however, varies across disciplines. Psychologists conceive it as a stage of identity development, wherein only oneself knows about one's sexuality (Pachankis and Jackson 2023). Sociologists reject this, as such narrow boundaries obscure the complexity of experiences within the closet (Patel 2024). They instead define it as a social condition that constrains the choice of coming out, emphasizing its macro‐ and meso‐level enforcement (Seidman 2002). Thus, individuals pressured or expected to conceal by broader social structures are considered closeted.

### 2.1 From a Closeted to Post‐Closeted Era

The metaphoric closet emerged as a social reality in response to the post‐war moral panic surrounding homosexuality, which “demonized” and systematically excluded queer people from participating in “respectable society” (Seidman 2002, 28). The suppression of homosexuality through state‐enforced legislation and policing, coupled with heterosexual domination, drove people into the closet (Seidman 2002). Seidman (2002, 29) refers to this outcome as a “historically specific social pattern” arising from temporal societal conditions. In Canada, the government perceived homosexuality as a national security threat and thus deployed Operation Fruit Machine in the 1950s–60s to purge all suspected gays and lesbians from the civil service (Kinsman 1995). During this time, many LGBTQ+ people concealed their identities to avoid the consequences of job loss and social exile, which would negatively affect their life chances (Williams et al. 2009). Thus, the closet originated as a safety mechanism.

Scholars suggest that the importance of the closet in people's lives has diminished since the 1980s, heralding what Seidman (2002) terms a post‐closeted era. This shift is attributed to improving societal attitudes toward homosexuality (Andersen and Fetner 2008) and progressive legal reform (Chauncey 2004), which have reduced the costs associated with coming out and enabled people to embrace queer lives publicly (Seidman 2002; Williams et al. 2009). The Western gay liberation movement concurrently championed out and proud ideologies (Staggenborg and Ramos 2016), creating sexual expectations of outness.

In a post‐closeted era, coming out is framed as healthy (Cole et al. 1996; Downs 2005; McLean 2007), politically responsible (Burgess 2005; Corrigan and Matthews 2003; Gross 1991), and an empowering act of identity liberation, in which there is “no moral alternative but to come out” (Rasmussen 2004, 146). Conversely, closeted people are perceived as unhealthy, politically irresponsible (Adams 2010), deficient, and ashamed of their sexuality (Meyer and Dean 1998; Rasmussen 2004). As such, Western LGBTQ+ communities now “vilified the closet” (Seidman 2002, 29) and hold an “attitude of contempt for people who are closeted” (Chee 2006, 114). In other words, being closeted is considered substandard in a post‐closeted era. Perspectives like these negate the intersecting cultural impediments that prevent queer Asian diaspora from fully coming out to their ethnic communities (Nadal 2021; Patel 2019; Siraj 2018).

### 2.2 The Closet as a Place of Safety?

Scholarship on closet‐related violence is scarce, focusing mainly on blackmailability—that is, threats from peers (Boucai 2022) or intimate partners (Kilday and Nash 2017) to out someone to their family or workplace. Blackmail exploits compromised secrecy, leveraging one's vulnerable social position for personal gain. Accounts of blackmail trace back to the 18th century (Kilday and Nash 2017), albeit predominantly against gay men in Western nations. While many closeted LGBTQ+ individuals fear being outed (Pachankis 2007), blackmail is only potent when homophobic social and legal structures exist (Kilday and Nash 2017). Hence, scholars frame vulnerability to blackmail as a product of these structures rather than an inherent feature of the closet (McLaren 2002; Sedgwick 1985). Accordingly, it is less prevalent in a post‐closeted era of progressive societal attitudes and legislation, mirroring the decline in blackmailability research in Western society over the past four decades.

Literature rarely considers closet‐related violence since transitioning to a post‐closeted era. Instead, scholarship generally assumes it is a place of safety. Hence, scholars frequently describe the closet as a “place of security and safety for the homosexual's vulnerable identity” (Mohler 2000, 49), “protection from homophobia” and “emotional, financial, and/or physical violence” (Choudhury 2020, 128), and a “safety zone” that provides a temporary escape from pervasive heterosexual ideologies in one's ethnic culture (Fisher 2003, 180). Empirical studies also replicate this presumption. Several quantitative studies identify concerns about acceptance, fear of rejection, and anticipation of stigmatizing reactions as motivations for sexual identity concealment (Jackson and Mohr 2016; Meidlinger and Hope 2014). Likewise, many qualitative studies identify a desire to avoid discrimination as a reason for remaining closeted (Bry et al. 2017; Feinstein et al. 2020). These reasons for concealment create an implicit paradox that positions harm and violence as a consequence of coming out and the closet as protection against it, reproducing the dominant notion of the closet as a place of refuge. By identifying this conjecture, I do not deny its function as a protective mechanism for LGBTQ+ people. Instead, I contend that it inadvertently overlooks the conceptual possibility of the closet as a site of violence.

### 2.3 Divergent Demands for Sexual Identity

Ross (2005) argues that the conceptual application of the closet is frequently removed from racial analysis, rendering it insufficient for understanding queer racialized realities. An intersectionality perspective can help consider how the interaction between multiple social location discourses informs one's identity (Crenshaw 1991). For diasporic subjects, conflicting cultural values of Western individualism and Eastern familialism produce divergent demands for articulating sexuality (Patel 2019). While coming out is considered an integral aspect of identity formation in Western LGBTQ+ cultures (Greene 1996; Mohler 2000), doing so can subject queer people of color to violence from their ethnic communities (Adur 2018; Ross 2005).

The South Asian community in Canada is diverse, encompassing individuals with ancestral roots in Afghanistan, Bangladesh, Bhutan, Burma, India, Maldives, Nepal, Pakistan, Tibet, Tamil Eelam, and Sri Lanka, as well as Indo‐Caribbeans, Indo‐Africans, and Fijians. They practice a variety of faiths, including Buddhism, Christianity, Catholicism, Hinduism, Islam, Jainism, and Sikhism (Fleras and Elliott 2007). Despite their heterogeneity, collectivist cultural values and expectations remain consistent across South Asian diasporas (Navsaria 2022; Zaidi et al. 2014) and are prioritized in raising second‐generation children (Maiter and George 2003).

Coming out poses significant risks for South Asians due to their collectivist cultural contexts (Islam 1998; Jaspal and Siraj 2011; Patel 2019). Families play a significant role in their child's identity formation (Siraj 2018). South Asians are expected to maintain familial honor through adherence to traditional gender roles, family obligations, and collectivist values regarding intimate relationships (e.g., marriage, heterosexuality, and chastity) (Bacchus 2017; Jaspal 2012; Siraj 2018; Yip 2004). Women are disproportionately responsible for upholding cultural traditions (Dasgupta 1998; Inman et al. 2001), as their behaviors reflect the family's collective integrity (Inman 2006). Any deviation from ethnocultural norms would risk tainting their family's reputation (Badruddoja 2008; McKeown et al. 2010). Upholding honor becomes complicated for QSAW, as their sexuality contests dominant expectations of heteronormative marriage (Gilbert et al. 2004; Siraj 2018). Thus, coming out threatens familial honor (Jaspal and Siraj 2011; Patel 2019; Yip 2004), potentially jeopardizing familial relations and parents’ ties within the diasporic community (Bacchus 2017; Jaspal 2012). In light of these culturally unique factors, QSAW may choose to remain closeted out of respect for their parents (Bacchus 2017; Jaspal and Siraj 2011; Siraj 2018; Yip 2004).

Many QSAW embrace double living (Islam 1998; Patel 2019) as a strategy for managing their identity in the closet (Boucai 2022). A double life involves separating one's “secretive homosexual life” from one's “conventional public heterosexual life” (Seidman 2002, 49). It allows LGBTQ+ people to engage in queer sociability and intimacy without fully coming out (Lo 2023; Svensson and Strand 2023). QSAW often live a double life by rejecting rigid identity labels to easily shift between identities in different situational and relational contexts (Narváez et al. 2009; Patel 2019), presenting traditional South Asian femininity (e.g., long hair) (Islam 1998; Patel 2019), rejecting identifiable queer signals (i.e., rainbows) (Patel 2021), and engaging in clandestine relations (Adur 2018; Bacchus 2017). Double living allows QSAW to meet ethnic expectations of heterosexuality (Bacchus 2017; Siraj 2018), thus preserving familial honor. However, doing so subjects them to undue pressure from their white counterparts to come out to their parents (Badruddoja 2008)—a dynamic I have conceptualized as culturally specific racism against QSAW within LGBTQ+ communities (see Patel 2019). Therefore, I predict that the convergence of Western coming‐out norms with ethnic expectations of concealment can exacerbate one's vulnerability to violence when navigating the closet.

## 3 Methods

This article seeks to understand the experiences of QSAW in the closet. I limited the sample to second‐ or 1.5‐generation millennials (born between 1981 and 1996), who share a similar immigrant family background. I selected millennials because this cohort came of age during the post‐closeted era, experiencing a similar national context of sexual minority rights and acceptance. I excluded Generation Z, as more than half were under the age of 18 at the time of this study.

I further restricted the sample to those who self‐identify as closeted, enabling a broader conceptual understanding of the closet and QSAW's experiences within it. Although South Asians comprise the largest ethnic minority population^3^ in Canada (Statistics Canada 2017a, 2017b), they remain the least represented racial group in the country's LGBTQ+ contexts—a disparity I documented in my previous study (Patel 2021). This makes QSAW's experiences imperative to investigate. Additionally, Canada is ranked among the most LGBTQ+‐friendly countries in the world (Bloom 2023), reducing the confounding effects of an unsafe national context on how QSAW experience the closet.

### 3.1 Data and Analysis

The data for this study includes 40 in‐depth interviews collected between June and September 2023. This was an optimal time for data collection, as Pride Festivals held across various Canadian cities during this period brought closet‐related strife and emotion to the cognitive forefront for QSAW (Patel 2019, 2021), enhancing memory recall and the accurate sharing of lived experiences in interviews.

I used snowball sampling to recruit participants. While this strategy relies on social networks, it is optimal for reaching populations that are hidden due to sensitivity and discrimination reasons and is especially effective in sexuality studies (Ghaziani 2014). Given that this study examines closeted individuals, snowball sampling is highly effective. I gained initial access to participants by digitally circulating a participant recruitment advertisement through the Queer South Asian Women's Network, a Canadian non‐profit organization with a large and established following of this hidden and difficult‐to‐access population (Patel 2021). Another initial access strategy I used involved asking people in my network to share information about my study through word‐of‐mouth with their respective social circles (cf. Browne 2005). Respondents recruited through snowball sampling contacted me directly through email to coordinate an interview time.

The final dataset consists of 40 interviews with QSAW in Canada. Within that sample, 31 identified as cisgender, and nine identified as gender non‐conforming. Given that QSAW prefer multiple labels or none (Patel 2019), I asked participants to share their identity labels and attraction. Seventeen respondents reported a bisexual attraction, and they identified with several possible labels, including bisexual, pansexual, demisexual, questioning, and/or queer. Twenty‐three participants reported lesbian attraction; they identified with one or more of these labels: gay, lesbian, and/or queer. Further, 17 participants were born in Canada, three were born in the United States, and 20 were born in Asia or Africa. All participants arrived in Canada with their families prior to the age of 14; the average age of arrival was 6.6 years old. Additionally, 28 participants currently reside in Ontario, eight in British Columbia, three in Alberta, and one in Quebec. In terms of the level of closetedness to family, 22 reported total non‐disclosure (i.e., not out to both parents), five reported that one parent (often the mother) knows but denies their sexuality, and 13 reported that both parents are in denial despite disclosure. Parental denial of a child's sexuality encompassed dismissal, disbelief, and pretending not to know—in all cases, the parent(s) maintained a false belief that their child is heterosexual (Patel 2024). See Table 1 for detailed demographic information.

**TABLE 1:** Participant demographics.

Pseudonym	Age	Ethnicity	Family religion	Occupation	Degree of closetedness to Family
Aditi	26	Bengali	Muslim	Graphic designer	Dad denied, mom unaware (both now deceased), not out to sibling
Maitreyi	26	Eelam Tamil	Catholic and Hindu	Student	Not out to parents or siblings
Ambika	26	Eelam Tamil	Hindu	Unemployed	Parents in denial
Devika	26	Eelam Tamil	Hindu	Security	Not out to parents, out to siblings
Satya	26	Eelam Tamil	Hindu	Student	Parents in denial
Vaishnavi	26	Eelam Tamil	Hindu	I.T.	Not out to parents, out to siblings
Gauri	26	Gujarati	Hindu	Student	Not out to parents
Nalini	26	Nepali	Buddhist	Unemployed	Not out to parents or siblings
Uma	26	Punjabi	Sikh	Doula	Not out to parents or sibling
Vaani	26	Punjabi	Sikh	Student	Not out to mom, dad is deceased, out to siblings
Parvati	27	Indian Tamil	Muslim	Politics	Not out to parents or sibling
Durga	27	Indo‐African Gujarati	Hindu	Unemployed	Mom in denial, not out to dad, out to sibling
Tara	27	Pakistani	Muslim	Engineer	Not out to dad, mom in denial, out to siblings
Deepa	28	Gujarati	Hindu	Music Artist	Parents in denial, out to sibling
Kamakshi	28	Gujarati	Hindu	HR	Parents in denial, out to siblings
Sati	28	Indo‐African Gujarati	Muslim	Unemployed	Not out to parents or siblings
Shitala	29	Eelam Tamil	Hindu	Data analyst	Not out to parents or brother, out to sister
Yogini	29	Pakistani	Muslim	Educator	Not out to parents or siblings
Shiva	29	Punjabi	Sikh	Unemployed	Parents in denial, out to sibling
Abirami	29	Sinhalese	Buddhist	Engineer	Parents in denial, out to sibling
Shreeja	30	Bengali	Muslim	Non‐profit	Not out to parents or sibling
Rukmini	30	Fijian	Hindu and Catholic	Non‐profit	Parents in denial, out to sibling
Jaya	30	Punjabi	Sikh	Professor	Not out to mom, dad is deceased
Neela	31	Pakistani	Muslim	Marketing	Mom in denial, dad is deceased, out to siblings
Saranya	32	Punjabi/ Kannada	Hindu	Teacher	Parents in denial, out to siblings
Saraswati	33	Fijian	Hindu	Counsellor	Parents in denial, out to siblings
Kumari	33	Nepali	Hindu	Filmmaker	Mom in denial, not out to dad
Lakshmy	34	Eelam Tamil	Christian	Social worker	Mom in denial, not out to dad, out to some siblings
Kaali	34	Eelam Tamil	Hindu	Police officer	Parents in denial, out to siblings
Ashanti	34	Punjabi	Hindu	I.T.	Not out to parents, out to sibling
Divya	35	Eelam Tamil	Hindu	Social worker	Not out to dad, mom is deceased, out to sibling
Tarini	35	Eelam Tamil	Hindu	Nurse	Not out to parents or siblings
Radha	35	Pakistani	Muslim	Non‐profit	Not out to parents or siblings
Vidya	35	Punjabi	Sikh	Teacher	Parents in denial, out to siblings
Shakti	36	Guyanese	Hindu and Christian	HR	Mom in denial, out to dad
Sridevi	39	Indian Tamil	Hindu	Educator	Not out to parents or sibling
Ganga	39	Punjabi	Sikh	Unemployed	Parents in denial
Bhavani	40	Punjabi	Hindu	Doctor	Not out to parents or siblings
Sita	42	Indo‐African Gujarati	Hindu	Administration	Not out to dad, mom is deceased
Bhumi	42	Indo‐African Gujarati	Muslim	Doctor	Not out to dad and brother, mom is deceased, out to sister

All participants provided informed consent prior to the interview. The interviews ranged from 32 to 117 min, averaging 61 min. Respondents could choose to participate in one‐on‐one interviews either through encrypted online Zoom software or in person. Only one respondent selected the latter at a remote location of their choice. Conducting interviews through Zoom offered cost‐effectiveness, convenience, and access to a nationwide sample. Recent studies affirm the acceptability of Zoom for qualitative data collection, ranking it on par with alternative interviewing tools, including face‐to‐face, telephone, and other video call platforms (Archibald et al. 2019). To develop rapport and ensure the comfort of the participants, I began each interview by disclosing my LGBTQ+ South Asian identity (cf. Patel 2019) and explaining my personal investment in this study as a community insider (cf. Bartholomay and Pendleton 2023). I asked questions related to the following themes: (1) the role of family in one's queer journey, (2) identity concealment reasons and strategies, and (3) experience of queer dating, hookups, and LGBTQ+ community events. I transcribed each interview and used NVivo to analyze the data line‐by‐line. To understand QSAW's motivations for, and experiences of, being closeted, I used an abductive approach (Timmermans and Tavory 2012, 173), an analytical strategy that sensitizes researchers to “surprises” in the data. Surprises are observations that counter implicit assumptions, expectations, and existing theories (Miles and Huberman 1994). During initial coding, I remained open to surprises (cf. Ghaziani 2024). I organized the data into codes related to experiences of violence in different relational contexts (i.e., parent‐child, romantic dyads, and LGBTQ+ peer relationships), the perceived motives for violence (i.e., expectations), navigation strategies, and impacts of these experiences. Following this, I engaged in code reduction. My initial stage of coding generated 62 codes. I thematically grouped these into eight organizing categories that revealed key subthemes (cf. Attride‐Stirling 2001). Each category described an aspect of the participants’ experience in the closet (e.g., family violence). I checked the empirical findings of each subtheme against literature on the closet and concealment to determine if and how they challenge existing theories and implicit assumptions. This article reports only five of these subthemes (see Table 2).

**TABLE 2:** Codes related to QSAW's closet experiences.

Code	How do QSAW experience the closet?	Agreement score
Family violence	Family uses violence to enforce the closet.	91.87%
Stressor from South Asian culture	Family imposes expectations for articulating sexuality and pressures to conform to heterosexual norms.	89.48%
Lateral LGBTQ+ violence	LGBTQ+ community uses micro‐aggressive violence to contest the closet and impose expectations of outness.	94.36%
Dating violence	QSAW are vulnerable to intimate partner violence.	97.42%

### 3.2 Reliability, Reflexivity, and Validity

One concern affecting qualitative research is the issue of reproducibility among coders (Campbell et al. 2013). I address this concern using a two‐step process: first, I engaged in code reduction, which is described above. Second, I conducted intercoder reliability tests to ensure the reproducibility of my codes. I did so by explaining my codebook to a research colleague, who is unaffiliated with my study, and removed all identifiers on the transcripts, leaving only uncoded blocks of highlighted text (Campbell et al. 2013). Using the coding scheme provided, the independent researcher then coded the eight organizing themes in 5% of my randomly selected data. All eight themes received agreement scores above 89.48%, and an average score of 93.95%, indicating excellent intercoder reliability.

As an insider of the population under inquiry, it is critical to discuss my reflexivity as a researcher. Reflexivity recognizes that the researcher's orientations are “shaped by their socio‐historical locations, including the values and interests that these locations confer upon them” (Hammersley and Atkinson 2007, 15). Accordingly, my social reality has been constructed by my lived experience as a diasporic South Asian lesbian. Acknowledging my proximity to this research, I made deliberate efforts to remain objective throughout the data collection and analysis process. To ensure the representativeness of my findings, I sought “unpatterns” (Miles and Huberman 1994, 263) by coding outliers and negative evidence (Miles and Huberman 1994, 271) under the same code as a given pattern, rather than creating separate codes. For example, I coded instances of supportive romantic partnerships under the *unhealthy queer relationship* code. Doing so allowed me to determine the significance of a pattern while taking outliers into account. Additionally, coding for negative evidence allowed me to paint a comprehensive picture of a given pattern, which was especially helpful for confirming findings in cases where participants elicited self‐contradictions. For instance, a few respondents initially expressed parental acceptance but later described parental actions that indicated otherwise (e.g., family violence).

## 4 Results: Violence Within the Closet

Whereas scholars generally assume the closet is a place of safety, I propose that it also operates as a site of violence. This section explores how families, LGBTQ+ peers, and intimate partners use violence to enforce, contest, or exploit the closet. The contradictory nature of ethnic expectations to stay closeted and sexual pressures to come out exacerbates QSAW's vulnerability to violence in the closet.

### 4.1 Family Violence

South Asian parents enforce the closet by imposing normative expectations of heterosexuality and using violence to suppress any deviation. Nearly all QSAW whose parents suspected or learned of their non‐heterosexuality were subjected to some degree of emotional, verbal, financial, and/or physical violence as an attempt to repress their queer tendencies. Emotional abuse is the most prevalent form of violence that parents, often mothers, inflict upon QSAW. This includes remarks such as, “We did so much for you; how could you do this to us?”; “You are so selfish, you're not thinking about anyone else”; “Don't come to our funeral”; and “Do you want us to die?,” as well as threats to commit suicide if their child continues to be queer, which was often said by South Asian mothers. Abuse can also manifest in non‐queer contexts. Saranya (lesbian, Kannadiga/Punjabi, age 32) describes instances of being denied food under her parents’ roof as a product of “misplaced anger” and “misplaced disappointment” stemming from her deviation from the expected heteronormative life path.

Parents also deployed financial abuse to enforce the closet. Shiva describes a life‐altering experience of financial abuse:

[My mom] used credit cards in my name that I didn't know about to damage my credit score, so I had to pay like thousands of dollars, because they were trying to send me to court for it. So, I called, and I was like, I don't know what this is, and […] the charges were like, $10,000 or something. And I'm just a university kid. I don't know, like, where this charge is coming from. And then I found out like, oh, it's for my parents, like, they did it for their business. So, then I found out that they weren't paying it back. My mom was like, ‘well, you could not, like, be gay, and we can pay this off for you. You can live a nice life with us. Or you can have all this debt to your name, and have a bad credit because of this, so you can't go anywhere or do anything on your own.’

Shiva's experience illustrates how parents leverage economic control to coerce compliance with heteronormative expectations. For Devika (lesbian, Eelam Tamil, age 26), financial violence gradually escalated from her parents requesting part of her income to forcing her to cover recurring expenses for their investment property's mortgage and shared vehicle she cannot use. This leaves Devika with little money and financially dependent on parents who regularly perpetrate various forms of violence.

Physical abuse is another prevalent means by which South Asian parents attempt to suppress their child's queerness. This includes slapping, hitting, and choking. Kaali (lesbian, Eelam Tamil, Hindu, age 34) describes a dispute with her dad after he learned she attended Pride in Toronto, leading him to suspect she is queer:

I came home to my room being demolished. Pictures being ripped apart. […] My picture with [ex‐girlfriend] was taken down. […] Then, my dad gets in my face yelling, like, ‘you need to change, you can't be like this, why are you doing this to us? We did all this stuff for you, and this is what you're gonna do?’ […] My dad did hit me like once or twice, and he was just like, ‘you can't do this shit kind of thing.’ […] I took a knife, and I was like, ‘would you rather, like, me kill myself or be gay?’ Like, I took the knife and put it to my stomach, and I was like, if this is what you want, then I'll just kill myself.

Kaali's mother intervened before the situation escalated. Her father has since adopted a “don't ask, don't tell” approach, in that he does not ask about her sexuality, nor does she mention it. Attendance at Pride, even under the guise of an ally, often led to violent familial encounters for QSAW, as parents feared their children would be seen on the news by their ethnic communities. Thus, even proximity to LGBTQ+ initiatives can increase QSAW's vulnerability to family violence.

QSAW exhibiting masculinity in their family household often experienced violence due to their gender expression. Satya (lesbian, Eelam Tamil, Hindu, age 26) describes her father's response when she attempted to cut her hair:

During the pandemic, I couldn't get a haircut, and so I took my father's clippers, and I shaved my head, and he like hit me, and like, verbally abused me, and said things like, ‘oh, you're not a boy, you're a girl. Who do you think you are? You're not my child, don't you dare touch my shit again.’

Other forms of violence directed at masculine expression included pressure to wear dresses and makeup. Devika shares that her parents “ridicule” her masculine fashion. During her sister's wedding, Devika expressed a desire to wear a new “pant‐drape style sari,” but was told by her parents, “You have to wear a proper sari for the wedding or don't even bother coming.” Although Devika has never attempted disclosure to her parents, her embodied queer cues (i.e., masculine clothing) raise enough suspicion for her parents to retaliate with violent means of control. Durga (lesbian, Gujarati, Hindu, age 27) explains that South Asian parents use violence to enforce the closet when their children deviate from heteronormative expectations, as severing familial ties would produce the same social consequences as coming out within the ethnic kin structure:

In South Asian families, if your parents don't accept your sexuality, then they won't necessarily kick you out or cut off ties either because that would incite gossip. […] So then, family rejection literally means violence. […] If they don't like that you're gay, they will be violent and abusive until you do what they say.

Durga abruptly left her parents’ home during the COVID‐19 lockdown when the “violence became unbearable.” She thought it would sever familial ties. Instead, her mother insisted they lie to her father and extended relatives about the reason she moved out to avoid reputational damage and gossip in their ethnic community. For many participants, living independently reduced parental surveillance and, in turn, lessened the severity of violence. However, it did not absolve them of the demands to remain closeted.

Even when explicit forms of violence subside—whether temporarily or over longer periods—the closet remains enforced, making it virtually impossible for QSAW to come out of it fully. Nearly all participants attribute this to their parents’ anxieties about judgment from their ethnic community and fear of dishonoring the family. In this way, being closeted is not always a personal choice for QSAW but a condition imposed through ethnic kinship structures.

Thus far, the findings demonstrate that parents suspecting or knowing about their child's queerness leads to violent attempts of sexual repression to ensure the ethnic community does not find out. In this way, familial violence functions to enforce the closet.

### 4.2 Lateral LGBTQ+ Community Violence

LGBTQ+ community members use microaggressive violence to contest the closet and enforce conformity with Western‐normative expectations of outness. All participants who engaged in queer spaces report experiencing microaggressions targeting their closetedness. This lateral violence manifests as pressures to sever familial ties, demands for outness, and dismissals of culturally specific reasons for remaining closeted.

QSAW routinely encounter microaggressions from queer peers that demand coming out by severing ties with unaccepting family members, disregarding the cultural contexts that shape these relationships. Saranya (lesbian, Kannadiga/Punjabi, age 32) explains the infeasibility of this expectation:

There's this expectation that if it's a toxic [family] environment, then you must exit. But […] I would not be able to cut contact with my parents or family […] I would lose our entire world of people in that. It would force, like, my cousins to take sides. […] The situation with my parents is not ideal. But like, if I weigh out the pros and cons, it's better than cutting off contact. Or at least that's how I feel. And [white queer] people have questioned that choice.

Saranya's desire to preserve ties with unaccepting family members is met with judgment from her white queer peers, who also disparage her strategies for managing kin ties, such as boundary setting. Deepa (bisexual, Gujarati, Hindu, age 28) echoes this sentiment:

When I've explained to non‐South Asian [queer] friends of feelings that come up, like, ‘oh, I'm feeling unsafe.’ or like, ‘is this an environment that I can exist in?’ […] the suggestions I would get are like, ‘oh, just cut your family off if they make you feel that way.’ And I think that is a very black‐and‐white perspective. That doesn't fit Brown people at all.

Through micro‐aggressive recommendations to sever familial ties and cultural insensitivity toward their reasons for being closeted, white LGBTQ+ people effectively impose expectations of outness onto QSAW.

Saranya shares that her existence as a queer Brown person precipitates a line of invasive questions to determine if she is closeted: “The first question is always, well, what do your parents think? Or like, do your parents know? […] which is like, not always something I wanna share, ‘cause it can be a very personal question.” Intrusive questions and unsolicited advice about how to articulate sexuality properly are common experiences for QSAW. Radha (lesbian, Pakistani, Muslim, age 35), who strictly hides her sexuality from her parents and siblings for safety reasons, including a desire to avoid family violence, shares that her white counterparts frequently urge her to “just come out, and live your true life.” Instead of encouraging her to be safe, Radha's peers enforce a Western‐normative prescription for queer authenticity. Ambika (bisexual, Eelam Tamil, age 29) shares a similar experience with a white queer colleague:

At [LGBTQ+] camp, there were a lot of microaggressions. […] One person who shared a bunk with me in training basically said like, ‘oh you know, maybe you just don't try hard enough to be out. You need to be more overtly out.’ And I was like, no that's not really happening pal, nice try. But yeah, they basically pinned it on me and were like, ‘It's on you if [family] don't get it and screw them.’ And I was like, that's my family we're talking about; I can't just leave them in the dust. That's not the way family works – not the way I was raised, at least. It was weird to get that pushback from a co‐worker to be like, you're not out enough. And I was like yeah, I'm trying, trust me, but this is as out as I will be.

Ambika's attempts to assert the infeasibility of fully coming out are continually met with resistance from her colleague, who insists that disclosure to family, and severing ties if needed, is the correct way to express queerness. This resistance reflects an unwillingness to understand non‐Western perspectives for being in the closet, indicating that cultural insensitivity fuels micro‐aggressive violence. Thus far, the results demonstrate that queer communities inflict violence to contest the closet, while ethnic communities do so to enforce the closet.

### 4.3 Dating Violence

This study further finds that dating while closeted heightens QSAW's vulnerability to intimate partner violence (IPV), although it manifests differently in inter‐ and intra‐ethnic dyads. In inter‐ethnic queer relationships, culturally insensitive attitudes towards being closeted often led to IPV against QSAW. Of the seven QSAW coupled with white women, six experienced pressures to come out. These respondents indicated that white partners frequently personalized their refusal to disclose their relationship to family, disregarding reassurance of cultural expectations to stay closeted. Tara recalls a quarrel during which her white ex‐girlfriend told her, “I don't feel like I'm important enough in your life for you to come out.” As a result, she felt pressured to choose between her family and her girlfriend. Tara eventually terminated the relationship, as the risk of familial violence outweighed the benefits of coming out to them. Four of those six QSAW succumbed to their partner's insistence to come out to their parents, and one was outed to her mother by her white girlfriend, resulting in several years of family violence for all five. Thus, cultural insensitivity towards their reasons for being closeted exacerbates QSAW's vulnerability to violence from their white counterparts while in the closet.

Several QSAW in intra‐ethnic relationships report that, although South Asian partners are more likely to respect their need to stay closeted, abusive behaviors are highly prevalent. This includes gaslighting, manipulation, infidelity, emotional abuse, sexual violence, and physical violence. Vaishnavi (lesbian, Eelam Tamil, Hindu, age 26) explains her challenge of dating while closeted:

Toxic behaviors from partners are much more normalized, and I accepted it because that's what I thought it was supposed to be like. Almost like I let her use being closeted to justify and excuse her harmful behaviors towards me.

Satya similarly believes the closet “is often like an excuse for their behavior.” She shares her experience of sexual violence: “Being raped by my girlfriend, and um, I wasn't able to tell anyone or legally pursue her […] I wasn't able to speak about it because we are closeted.” Like Satya, being closeted prevented many QSAW from seeking support in the face of violence.

Tara, Durga, and Deepa believe that an inability to get a friend's or parent's opinion about their relationship made them more prone to accepting unhealthy behaviors from a partner. Durga describes how her ex‐girlfriend, who insisted on keeping their relationship a secret, exploited the closet to not only excuse her present infidelity but also to justify future acts of it:

[Ex‐girlfriend] withheld affection and said it was for her safety, but then she would be very touchy and flirty with other girls. Then I found out she had sex with my friend when we were dating, and I was like, what the fuck? She got mad at me for not letting her express herself, and that her family makes her repress her sexuality, and I'm doing the same thing by asking her not to cheat on me.

Durga initially believed keeping the violence a secret was important for protecting their safety as closeted individuals; however, she felt conflicted after her ex‐girlfriend “threatened to sue me for outing her if I told anyone about her hitting me.” Vaishnavi, Kaali, Durga, and Satya believe IPV occurs in intra‐ethnic dyads due to the known cultural expectation of staying in the closet, which safeguards the abuser from reputational damage. As Vaishnavi puts it, “they act toxic because they know you can't tell anyone.” These instances demonstrate that the need to hide one's own and/or their abuser's sexual identity exacerbates QSAW's vulnerability to IPV in the closet.

These findings suggest a racialized pattern in the motives for violence. White partners and queer communities largely inflict violence because QSAW are closeted, enforcing sexual expectations of outness. South Asian families and partners do so for reasons related to secrecy. Specifically, parents use violence to enforce sexual secrecy (i.e., stay closeted), while partners exploit the closet's condition of secrecy to enact abusive behaviors with impunity.

QSAW's experiences of violence in the closet from family members, LGBTQ+ peers, and intimate partners negatively impact their well‐being. The respondents often shared their experiences in conjunction with their emotional impact, noting that it elevated feelings of depression, anxiety, PTSD, chronic stress, and suicidal ideation. Satya shares, “In [my] first year [of university], I had attempted suicide like 8 times back‐to‐back, because I couldn't go on living a double life.” Many QSAW reported attempting suicide at least once in their lifetime due to the stressful experiences stemming from these incongruent expectations to be out or remain closeted. To cope with these challenges, participants recount finding relief in community spaces dedicated to QSAW.

## 5 Discussion and Theoretical Implications

While scholars have established that the closet persists during the post‐closeted era, particularly in workplaces (Orzechowicz 2016; Williams et al. 2009) and collectivist families (Patel 2024), the possibility of violence within it has been gravely overlooked. The data present strong evidence of the closet's dual function as a site of safety and violence. Whereas being in the closet protects individuals from unfavorable outcomes, such as ostracization from their ethnic communities (Bacchus 2017), loss of familial honor (Patel 2024), and queerphobic discrimination (Suppes et al. 2021), it concurrently exacerbates their vulnerability to violence from their family, the LGBTQ+ community, and intimate partners.

**FIGURE 1:**
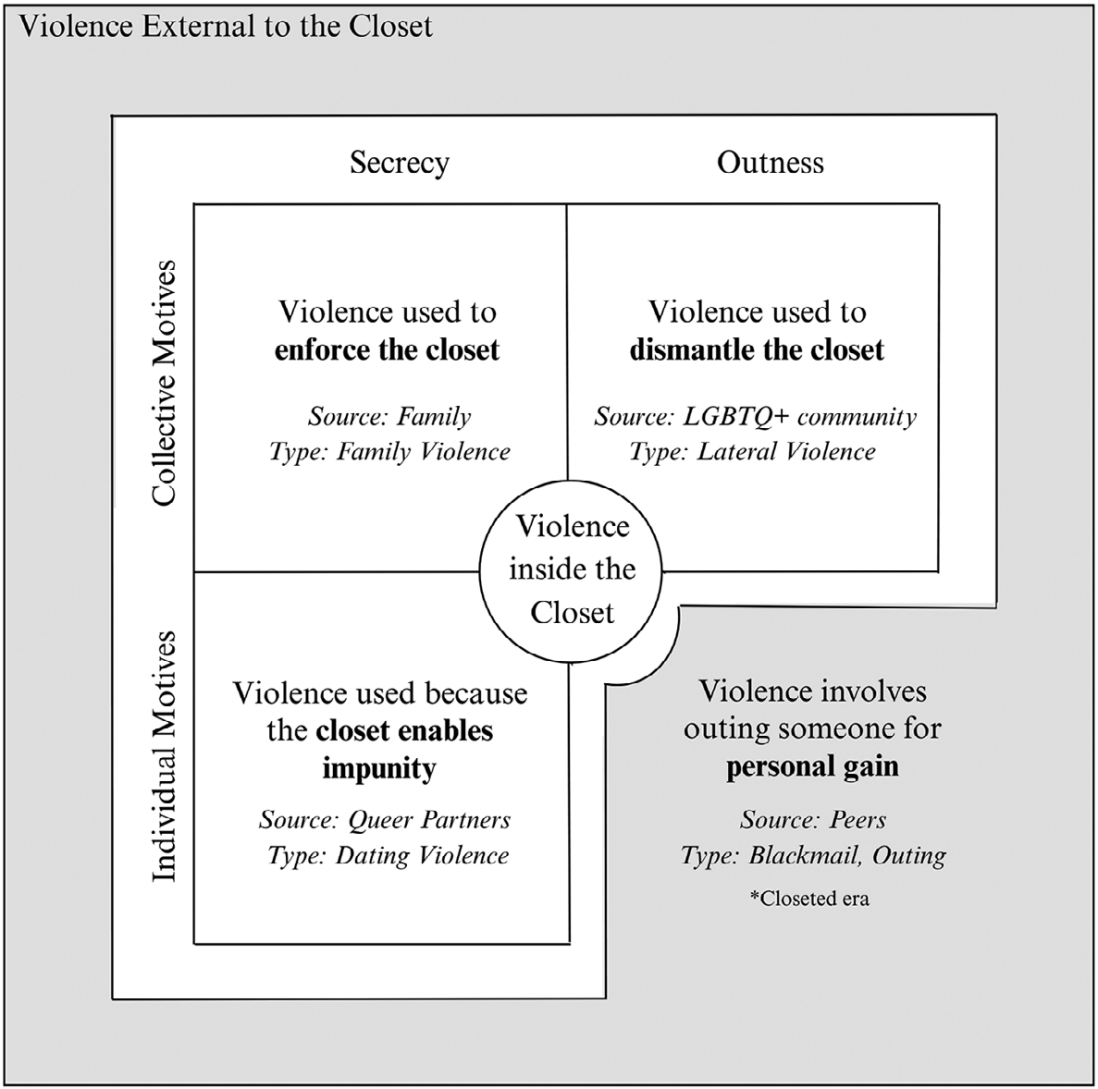
Typological model of violence in the closet.

In ethnic kin structures, closeted individuals are those who cannot be out to their ethnic communities and extended families (Patel 2024). Their revelation of identity to trusted others, such as partners, queer peers, parent(s), and friends, is thus considered an invitation into one's closet. Parents who suspect their child's non‐heterosexuality and suppress it using violence are considered self‐invited into the closet, as they aim to enforce secrecy from the broader ethnic community. Violence is not inflicted arbitrarily; rather, it is perpetrated by the partner(s), queer peers, and parent(s) whom QSAW invited into their closet. These individuals deploy violence as a strategic tool to regulate the way QSAW navigate their closetedness. Figure 1 summarizes the functions, forms, and sources of violence into a typological model of violence inside the closet.

In the post‐closeted era, family violence serves to enforce the closet. South Asian parents deploy violence to repress their child's queerness and enforce conformity to heteronormativity, preserving familial honor in their ethnic community. This includes emotional, verbal, physical, and financial violence. Parents may target their child's economic resources to create financial dependency, making it easier to regulate their child's sexual identity and restrict their autonomy. Violence is thus a strategic tool to keep QSAW in the closet.

The transition into a post‐closeted era created conditions for lateral queer violence within the closet, as outness became the normative expectation and being closeted was vilified (Rasmussen 2004; Seidman 2002). Driven by a broader cultural imperative to dismantle the closet, LGBTQ+ community members and white intimate partners perpetrate violence against closeted individuals. This includes microaggressions that relentlessly enforce coming out, prescribe individualistic sexual trajectories involving severing familial ties, and egregiously disregard the culturally specific reasons QSAW must remain closeted.

Intra‐ethnic queer intimate partners may enact abusive behaviors because the closet's quality of secrecy enables impunity. Unlike family and LGBTQ+ community members, who use violence in interpersonal relationships to achieve collective goals (i.e., enforce or contest the closet), intimate partners exploit the closet for their own benefit. Addressing a scholarly gap identified by Lo (2023), I find that dating while closeted makes QSAW more prone to experiencing and accepting intimate partner abuse. QSAW have little recourse when abuse occurs, as seeking help can risk outing both parties, further endangering their safety. Some abusers explicitly forbid their partner from informing others about the violence under the guise of outing, thus regulating how they navigate the closet. Therefore, the closet is a site of violence precisely because of the secrecy that made it safe in the first place.

Family violence, lateral queer violence, and IPV transpire concurrently in the closet but can also occur independently. All participants described experiencing at least two of these, mostly from their family and queer peers. These forms of violence reinforce one another, as they embody competing normative expectations (i.e., outness or closetedness). This means QSAW will continue enduring multiple forms of violence in the closet as long as contradictory expectations persist. This demonstrates the intersectional complexities of experiences in the closet in a post‐closeted era.

The explication of violence within the closet has critical implications for research on the closet and post‐closeted era. Contrasting the findings with extant literature, I distinguish two types of violence toward closeted people: violence inside the closet, which is unrelated to outness and perpetrated by those invited into one's closet, and violence external to the closet, which involves or threatens outing, with harm enacted by external structures as a result. Scholars argue that blackmail and outing are made possible by the state's legitimation of the closet and imposition of consequences for being out (e.g., job loss). Although the closet continues to be enforced in the post‐closeted era, albeit by the ethnic kin structure, the violence closeted people experience differs substantially. The participants in this study did not report blackmail or outing, which is the traditional conceptualization of harm related to the closet. The one participant who did report being outed caveated that it served to reinforce the closet, qualifying it as violence inside. This indicates a shift in the location of violence: in the closeted era, harm was contingent upon leaving the closet, whereas in a post‐closeted era, violence occurs within the confines of it.

The presumption of the closet as a place of safety is reinforced by scholarly claims that LGBTQ+ individuals who are more out experience greater discrimination (Choudhury et al. 2009; Suppes et al. 2021). This argument holds because people with greater outness have more social interactions where their identity is known and, thus, are more likely to be targeted than someone who hides their sexuality. However, it falsely and damagingly implies that closeted individuals are less likely to experience harm because, as scholars note, the closet protects against discrimination (Suppes et al. 2021) and the stress of it (Pachankis et al. 2020). Moving forward, scholars should cogitate on closet‐specific forms of discrimination and violence.

The results of this study corroborate scholarly arguments that society has transitioned to a post‐closeted era (Seidman 2002). In fact, it is the normalcy of outness and cultural vilification of the closet that exacerbates closeted QSAW's vulnerability to lateral violence from LGBTQ+ peers. It is thus unique to the current era, emerging because of improving public opinion of sexual minorities that normalizes outness (Keleher and Smith 2012). This indicates that queer people who are out uphold a social hierarchy subordinating closeted individuals. Scholars should account for this power dynamic when examining LGBTQ+ experiences in the post‐closeted era.

## 6 Conclusion

This article re‐theorizes the closet as a dual site of safety and violence. Through the experiences of 1.5‐ and second‐generation QSAW, I conceptualize three distinct forms of violence within the closet: family violence, lateral queer violence, and dating violence—each operating to enforce, contest, and exploit the closet. These are unique to the post‐closeted era; thus, I distinguish between violence inside the closet from that external to it. Violence within the closet has three defining characteristics: (1) it functions to regulate how someone navigates the closet, (2) it occurs within the confines of the closet, unrelated to outness or the threat of it, and (3) it is inflicted by those invited into one's closet.

This article subverts the assumption that the closet is solely protective. Instead, I demonstrate how family members, queer peers, and intimate partners use violence as a regulatory tool to enforce competing expectations of outness and staying closeted. Therefore, I argue that being closeted exacerbates QSAW's vulnerability to violence.

The results are significant in two ways. First, I destabilize the assumptions that the closet functions solely as a refuge by theorizing how violence operates within it. Second, I establish the intersectional complexities of the closet, showing how sexual expectations of outness and ethnic demands to remain closeted violently converge in the post‐closeted era. Together, these offer novel perspectives on the closet and its implications in Western nations.

The results yield urgent recommendations for practice. First, service providers (e.g., therapists) should withhold suggestions to come out to family and instead, validate lifelong concealment as a viable option. Second, homophilous social support is imperative for LGBTQ+ Asian diaspora, who often lack supportive family and a sense of belonging in broader queer spaces (Ching et al. 2018). To offset the emotional consequences of experiencing violence within the closet, I urge agencies to improve funding for organizations serving QSAW (Patel 2019).

### 6.1 Generalizability

The narratives herein are unique to QSAW yet broadly applicable to anyone who has two or more identities with conflicting expectations of normativity. Thus, the findings are generalizable to Asian, Hispanic, Middle Eastern, and Black LGBTQ+ diasporic groups, who have similar colonial, racial, and familial contexts (Gattamorta and Quidley‐Rodriguez 2018; Svensson and Strand 2023).

### 6.2 Limitations

A limitation of this study is that I did not employ quota sampling techniques to generate a sample with proportionate demographic characteristics (i.e., X number of participants per province, ethnicity, sexuality, etc.). Instead, I accepted all interested participants who met the study's eligibility criteria. I did so to offset the difficulty of recruiting participants from a hidden population and to recruit a larger sample size. The final sample is fairly proportionate in its ethnic and sexual composition; the only disproportionate characteristic is geographic location (e.g., 70% of respondents reside in Ontario). However, this limitation did not impact the quality of my results, as researchers have not documented that closet experiences vary as a function of province.

## Endnotes

1 Lesbian describes an identity or dyad with attraction to/between women or nonbinary people.

2 Queer is an umbrella term that describes non‐heterosexual identities (Russell et al. 2023). I use it interchangeably with gay, lesbian, bisexual, and other non‐heterosexual identities.

3 Statistics Canada refers to ethnic minorities as “visible minority” when reporting population data.

## References

[cars70016-bib-0001] Adams, T. E. 2010. “Paradoxes of Sexuality, Gay Identity, and the Closet.” Symbolic Interaction 33, no. 2: 234–256.

[cars70016-bib-0002] Adur, S. M. 2018. “In Pursuit of Love: ‘Safe Passages’, Migration and Queer South Asians in the US.” Current Sociology 66, no. 2: 320–334.

[cars70016-bib-0003] Andersen, R. , and T. Fetner . 2008. “Economic Inequality and Intolerance: Attitudes Toward Homosexuality in 35 Democracies.” American Journal of Political Science 52, no. 4: 942–958.

[cars70016-bib-0004] Archibald, M. M. , R. C. Ambagtsheer , M. G. Casey , and M. Lawless . 2019. “Using Zoom Videoconferencing for Qualitative Data Collection: Perceptions and Experiences of Researchers and Participants.” International Journal of Qualitative Methods 18, no. 1: 1–8.

[cars70016-bib-0005] Attride‐Stirling, J. 2001. “Thematic Networks: An Analytic Tool for Qualitative Research.” Qualitative Research 1, no. 3: 385–405.

[cars70016-bib-0006] Bacchus, N. S. 2017. “Shifting Sexual Boundaries: Ethnicity and Pre‐Marital Sex in the Lives of South Asian American Women.” Sexuality & Culture 21, no. 3: 776–794.

[cars70016-bib-0007] Badruddoja, R. 2008. “Queer Spaces, Places, and Gender: The Tripologies of Rupa and Ronica.” National Women's Studies Association Journal 20, no. 2: 156–188.

[cars70016-bib-0008] Bartholomay, D. J. , and M. Pendleton . 2023. “Doing Sexuality: How Married Bisexual, Queer, and Pansexual People Navigate Passing and Erasure.” Sociological Quarterly 64, no. 3: 520–539.

[cars70016-bib-0009] Bloom, L. B. 2023. “20 Safest Countries for LGBTQ Travelers, Ranked in a New Report.” *Forbes*, March 7.

[cars70016-bib-0010] Boucai, M. 2022. “Topology of the Closet.” Journal of Homosexuality 69, no. 4: 587–611. 33399520 10.1080/00918369.2020.1851957

[cars70016-bib-0011] Browne, K. 2005. “Snowball Sampling: Using Social Networks to Research Non‐Heterosexual Women.” International Journal of Social Research Methodology 8, no. 1: 47–60.

[cars70016-bib-0012] Bry, L. J. , B. Mustanski , R. Garofalo , and M. N. Burns . 2017. “Management of a Concealable Stigmatized Identity: A Qualitative Study of Concealment, Disclosure, and Role Flexing Among Young, Resilient Sexual and Gender Minority Individuals.” Journal of Homosexuality 64, no. 6: 745–769. 27633070 10.1080/00918369.2016.1236574 PMC5352544

[cars70016-bib-0013] Burgess, S. 2005. “Did the Supreme Court Come out in Bush v. Gore? Queer Theory on the Performance of the Politics of Shame.” Differences 16, no. 1: 126–146.

[cars70016-bib-0014] Campbell, J. L. , C. Quincy , J. Osserman , and O. K. Pedersen . 2013. “Coding in‐Depth Semistructured Interviews: Problems of Unitization and Intercoder Reliability and Agreement.” Sociological Methods & Research 42, no. 3: 294–320.

[cars70016-bib-0015] Chauncey, G. 2004. “‘What Gay Studies Taught the Court’: The Historians' Amicus Brief in Lawrence v. Texas.” GLQ 10, no. 3: 509–538.

[cars70016-bib-0016] Chee, A. 2006. “Dick.” In From Boys to Men: Gay Men Write About Growing up, edited by T. Gideonse and R. Williams . Carroll and Graf.

[cars70016-bib-0017] Ching, T. H. W. , S. Y. Lee , J. Chen , R. P. So , and M. T. Williams . 2018. “A Model of Intersectional Stress and Trauma in Asian American Sexual and Gender Minorities.” Psychology of Violence 8, no. 6: 657–668.

[cars70016-bib-0093] Choudhury, P. P. 2020. “The Violence That Dares Not Speak Its Name: Invisibility in the Lives of Lesbian and Bisexual South Asian American Women.” In Body Evidence: Intimate violence against South Asian women in America, edited by S. Dasgupta , 126–138. Rutgers University Press.

[cars70016-bib-0018] Choudhury, P. P. , N. S. Badhan , J. Chand , et al. 2009. “Community Alienation and Its Impact on Help‐Seeking Behavior Among LGBTIQ South Asians in Southern California.” Journal of Gay & Lesbian Social Services 21, no. 2–3: 247–266.

[cars70016-bib-0019] Cole, S. W. , M. E. Kemeny , S. E. Taylor , and B. R. Visscher . 1996. “Elevated Physical Health Risk Among Gay Men Who Conceal Their Homosexual Identity.” Health Psychology 15, no. 4: 243–251. 8818670 10.1037//0278-6133.15.4.243

[cars70016-bib-0020] Corrigan, P. W. , and A. K. Matthews . 2003. “Stigma and Disclosure: Implications for Coming out of the Closet.” Journal of Mental Health 12, no. 3: 235–248.

[cars70016-bib-0021] Crenshaw, K. 1991. “Mapping the Margins: Intersectionality, Identity Politics, and Violence Against Women of Color.” Stanford Law Review 43, no. 6: 1241.

[cars70016-bib-0022] Dasgupta, S. 1998. “Gender Roles and Cultural Continuity in the Asian Indian Immigrant Community in the U.S.” Sex Roles 38, no. 11/12: 953–974.

[cars70016-bib-0023] D'augelli, A. R. , A. H. Grossman , and M. T. Starks . 2008. “Families of Gay, Lesbian, and Bisexual Youth: What Do Parents and Siblings Know and How Do They React?” Journal of GLBT Family Studies 4, no. 1: 95–115.

[cars70016-bib-0024] D'Augelli, A. R. , and S. L. Hershberger . 1993. “Lesbian, Gay, and Bisexual Youth in Community Settings: Personal Challenges and Mental Health Problems.” American Journal of Community Psychology 21, no. 4: 421–448. 8192119 10.1007/BF00942151

[cars70016-bib-0025] Downs, A. 2005. The Velvet Rage: Overcoming the Pain of Growing up Gay in a Straight Man's World. Perseus.

[cars70016-bib-0026] Doyle, D. M. , and M. Barreto . 2023. “Toward a More Relational Model of Sexual Minority Identity Concealment.” Archives of Sexual Behavior 52, no. 5: 1911–1916. 36443612 10.1007/s10508-022-02491-5 PMC10322775

[cars70016-bib-0027] Dutton, D. G. 2006. Rethinking Domestic Violence. UBC Press.

[cars70016-bib-0028] Feinstein, B. A. , C. D. Xavier Hall , C. Dyar , and J. Davila . 2020. “Motivations for Sexual Identity Concealment and Their Associations With Mental Health Among Bisexual, Pansexual, Queer, and Fluid (Bi+) Individuals.” Journal of Bisexuality 20, no. 3: 324–341. 33727893 10.1080/15299716.2020.1743402 PMC7958702

[cars70016-bib-0029] Fisher, D. 2003. “Immigrant Closets: Tactical‐Micro‐Practices‐in‐the‐Hyphen.” Journal of Homosexuality 45, no. 2–4: 171–192. 14651179 10.1300/J082v45n02_08

[cars70016-bib-0030] Fleras, A. , and J. L. Elliott . 2007. Unequal Relations: An Introduction to Race, Ethnic, and Aboriginal Dynamics in Canada. 5th ed. Pearson Prentice Hall.

[cars70016-bib-0031] Gattamorta, K. , and N. Quidley‐Rodriguez . 2018. “Coming out Experiences of Hispanic Sexual Minority Young Adults in South Florida.” Journal of Homosexuality 65, no. 6: 741–765. 28771094 10.1080/00918369.2017.1364111 PMC5797510

[cars70016-bib-0032] Ghaziani, A. 2014. There Goes the Gayborhood? Princeton University Press.

[cars70016-bib-0033] Ghaziani, A. 2024. Long Live Queer Nightlife: How the Closing of Gay Bars Sparked a Revolution. 1st ed. Princeton University Press.

[cars70016-bib-0034] Ghaziani, A. , and A. Holmes . 2023. “Distinguishing but Not Defining: How Ambivalence Affects Contemporary Identity Disclosures.” Theory and Society 52, no. 5: 913–945.

[cars70016-bib-0035] Gilbert, P. , J. Gilbert , and J. Sanghera . 2004. “A Focus Group Exploration of the Impact of Izzat, Shame, Subordination and Entrapment on Mental Health and Service Use in South Asian Women Living in Derby.” Mental Health, Religion & Culture 7, no. 2: 109–130.

[cars70016-bib-0036] Goffman, E. 1963. Stigma: Notes on the Management of Spoiled Identity. 1st ed. Simon & Schuster.

[cars70016-bib-0037] Gracia, E. , C. M. Rodriguez , M. Martín‐Fernández , and M. Lila . 2020. “Acceptability of Family Violence: Underlying Ties Between Intimate Partner Violence and Child Abuse.” Journal of Interpersonal Violence 35, no. 17–18: 3217–3236. 29294751 10.1177/0886260517707310

[cars70016-bib-0038] Greene, B. 1996. “Lesbian Women of Colour: Triple Jeopardy.” Journal of Lesbian Studies 1: 109–147. 10.1300/J155v01n01_09 24784950

[cars70016-bib-0039] Gross, L. 1991. “The Contested Closet: The Ethics and Politics of Outing.” Critical Studies in Mass Communication 8, no. 3: 352–388.

[cars70016-bib-0040] Hammersley, M. , and P. Atkinson . 2007. Ethnography: Principles in Practice. 3rd ed. Routledge.

[cars70016-bib-0041] Inman, A. G. 2006. “South Asian Women: Identities and Conflicts.” Cultural Diversity and Ethnic Minority Psychology 12, no. 2: 306–319. 16719579 10.1037/1099-9809.12.2.306

[cars70016-bib-0042] Inman, A. G. , N. Ladany , M. G. Constantine , and C. K. Morano . 2001. “Development and Preliminary Validation of the Cultural Values Conflict Scale for South Asian Women.” Journal of Counseling Psychology 48, no. 1: 17–27.

[cars70016-bib-0043] Islam, N. 1998. “Naming Desire, Shaping Identity: Tracing the Experiences of Indian Lesbians in the United States.” In A Patchwork Shawl: Chronicles of South Asian Women in America, edited by S. Dasgupta , 72–94. Rutgers University Press.

[cars70016-bib-0044] Jackson, S. D. , and J. J. Mohr . 2016. “Conceptualizing the Closet: Differentiating Stigma Concealment and Nondisclosure Processes.” Psychology of Sexual Orientation and Gender Diversity 3, no. 1: 80–92.

[cars70016-bib-0045] Jaspal, R. 2012. “‘I Never Faced up to Being Gay’: Sexual, Religious and Ethnic Identities Among British Indian and British Pakistani Gay Men.” Culture, Health & Sexuality 14, no. 7: 767–780. 10.1080/13691058.2012.693626 22651130

[cars70016-bib-0046] Jaspal, R. , and A. Siraj . 2011. “Perceptions of ‘Coming out’ Among British Muslim Gay Men.” Psychology and Sexuality 2, no. 3: 183–197.

[cars70016-bib-0047] Keleher, A. , and E. R. A. N. Smith . 2012. “Growing Support for Gay and Lesbian Equality Since 1990.” Journal of Homosexuality 59, no. 9: 1307–1326. 23101499 10.1080/00918369.2012.720540

[cars70016-bib-0048] Kilday, A.‐M. , and D. S. Nash . 2017. “From Blackmail and the Closet to Pride and Shame: Homosexuality and Identity—The Military Example.” In Shame and Modernity in Britain, 243–273, Palgrave Macmillan UK.

[cars70016-bib-0049] Kinsman, G. 1995. “‘Character Weaknesses’ and ‘Fruit Machines’: Towards an Analysis of the Anti‐Homosexual Security Campaign in the Canadian Civil Service.” Labour /Le Travail 35: 133.

[cars70016-bib-0050] Lawson, J. 2012. “Sociological Theories of Intimate Partner Violence.” Journal of Human Behavior in the Social Environment 22, no. 5: 572–590.

[cars70016-bib-0051] Lo, I. P. Y. 2023. “Violence in the ‘Double Closet’: Female Same‐Sex Intimate Partner Violence and Minority Stress in China.” Journal of Lesbian Studies 27, no. 1: 137–145. 35757991 10.1080/10894160.2022.2091732

[cars70016-bib-0052] Maiter, S. , and U. George . 2003. “Understanding Context and Culture in the Parenting Approaches of Immigrant South Asian Mothers.” Affilia 18, no. 4: 411–428.

[cars70016-bib-0053] Malley‐Morrison, K. , and D. A. Hines . 2012. Family Violence in a Cultural Perspective: Defining, Understanding, and Combating Abuse. SAGE Publications.

[cars70016-bib-0054] McKeown, E. , S. Nelson , J. Anderson , N. Low , and J. Elford . 2010. “Disclosure, Discrimination and Desire: Experiences of Black and South Asian Gay Men in Britain.” Culture, Health & Sexuality 12, no. 7: 843–856. 10.1080/13691058.2010.499963 20665298

[cars70016-bib-0055] McLaren, A. 2002. Sexual Blackmail: A Modern History. 1st ed. Harvard University Press.

[cars70016-bib-0056] McLean, K. 2007. “Hiding in the Closet? Bisexuals, Coming out, and the Disclosure Imperative.” Journal of Sociology 43, no. 2: 151–166.

[cars70016-bib-0057] Meidlinger, P. C. , and D. A. Hope . 2014. “Differentiating Disclosure and Concealment in Measurement of Outness for Sexual Minorities: The Nebraska Outness Scale.” Psychology of Sexual Orientation and Gender Diversity 1, no. 4: 489–497.

[cars70016-bib-0058] Meyer, I. H. , and L. Dean . 1998. “Internalized Homophobia, Intimacy, and Sexual Behavior Among Gay and Bisexual Men.” In Stigma and Sexual Orientation: Understanding Prejudice Against Lesbians, Gay Men, and Bisexuals, 180–186. SAGE.

[cars70016-bib-0059] Miles, M. B. , and M. A. Huberman . 1994. “Making Good Sense: Drawing and Verifying Conclusions.” In Qualitative Data Analysis, edited by M. B. Miles and A. M. Huberman , 245–287. Sage.

[cars70016-bib-0060] Mohler, M. 2000. Homosexual Rites of Passage: A Road to Visibility and Validation. Haworth Gay & Lesbian Studies. Harrington Park Press.

[cars70016-bib-0061] Nadal, K. L. 2013. That's so Gay! Microaggressions and the Lesbian, Gay, Bisexual, and Transgender Community. American Psychological Association.

[cars70016-bib-0062] Nadal, K. L. 2021. Filipino American Psychology: A Handbook of Theory, Research, and Clinical Practice. 2nd ed. Wiley.

[cars70016-bib-0063] Narváez, R. F. , I. H. Meyer , R. M. Kertzner , S. C. Ouellette , and A. R. Gordon . 2009. “A Qualitative Approach to the Intersection of Sexual, Ethnic and Gender Identities.” Identity 9: 63–86. 27683200 10.1080/15283480802579375 PMC5036453

[cars70016-bib-0064] Navsaria, N. 2022. “Considerations for South Asian American Parenting and Families.” In Counseling and Psychotherapy for South Asian Americans: Identity, Psychology, and Clinical Implications, 167–193. Routledge.

[cars70016-bib-0065] Orzechowicz, D. 2016. “The Walk‐In Closet: Between ‘Gay‐Friendly’ and ‘Post‐Closeted’ Work.” In Research in the Sociology of Work, edited by S. Vallas , vol. 29, 187–213. Emerald Group Publishing Limited.

[cars70016-bib-0066] Pachankis, J. E. 2007. “The Psychological Implications of Concealing a Stigma: A Cognitive‐Affective‐Behavioral Model.” Psychological Bulletin 133, no. 2: 328–345. 17338603 10.1037/0033-2909.133.2.328

[cars70016-bib-0067] Pachankis, J. E. , and S. D. Jackson . 2023. “A Developmental Model of the Sexual Minority Closet: Structural Sensitization, Psychological Adaptations, and Post‐Closet Growth.” Archives of Sexual Behavior 52, no. 5: 1869–1895. 35978203 10.1007/s10508-022-02381-w PMC9935753

[cars70016-bib-0068] Pachankis, J. E. , C. P. Mahon , S. D. Jackson , B. K. Fetzner , and R. Bränström . 2020. “Sexual Orientation Concealment and Mental Health: A Conceptual and Meta‐Analytic Review.” Psychological Bulletin 146, no. 10: 831–871. 32700941 10.1037/bul0000271 PMC8011357

[cars70016-bib-0069] Patel, S. 2019. “‘Brown Girls Can't Be Gay’: Racism Experienced by Queer South Asian Women in the Toronto LGBTQ Community.” Journal of Lesbian Studies 23, no. 3: 410–423. 30907270 10.1080/10894160.2019.1585174

[cars70016-bib-0070] Patel, S. 2021. “The Politics of (Not) Giving a Sh*T: Understanding the Invisibility of Queer South Asian Women in Pride Toronto.” Master's thesis, University of Ottawa.

[cars70016-bib-0071] Patel, S. 2024. “Theorizing a Denial Reaction to Coming out: Revising Goffman's Stigma Through a Sexual Identity Process Model.” Sociology Compass 18, no. 7: 1–16.

[cars70016-bib-0072] Ponse, B. 1976. “Secrecy in the Lesbian World.” Journal of Contemporary Ethnography 5, no. 3: 313–338.

[cars70016-bib-0073] Rasmussen, M. L. 2004. “The Problem of Coming Out.” Theory Into Practice 43, no. 2: 144–150.

[cars70016-bib-0074] Ross, M. B. 2005. “Beyond the Closet as Raceless Paradigm.” In Black Queer Studies: A Critical Anthology, edited by P. E. Johnson and M. G. Henderson , 161–189. Duke University Press.

[cars70016-bib-0075] Russell, S. T. , M. D. Bishop , and J. N. Fish . 2023. “Expanding Notions of LGBTQ+.” Annual Review of Sociology 49, no. 1: 281–296. 10.1146/annurev-soc-030320-032256 PMC11709134 39781443

[cars70016-bib-0076] Schäfer, J. 2024. “A Sociology of Violence: Violence Is Systemic and Relational.” In What Is Sexualized Violence?: Intersectional Readings, 178–204. Routledge.

[cars70016-bib-0077] Schmitz, R. M. , and K. A. Tyler . 2018. “Contextual Constraints and Choices: Strategic Identity Management Among LGBTQ Youth.” Journal of LGBT Youth 15, no. 3: 212–226.

[cars70016-bib-0078] Sedgwick, E. K. 1985. Between Men: English Literature and Male Homosocial Desire. Columbia University Press.

[cars70016-bib-0079] Sedgwick, E. K. 2008. Epistemology of the Closet. University of California Press.

[cars70016-bib-0080] Seidman, S. 2002. Beyond the Closet: The Transformation of Gay and Lesbian Life. Routledge.

[cars70016-bib-0081] Siraj, A. 2018. “British Pakistani Lesbians Existing Within the Confines of the Closet.” Culture, Health & Sexuality 20, no. 1: 28–39. 10.1080/13691058.2017.1323349 28508706

[cars70016-bib-0082] Smith, M. 2011. “Canada: The Power of Institutions.” In The Lesbian and Gay Movement and the State: Comparative Insights Into a Transformed Relationship, edited by M. Tremblay , D. Paternotte , and C. Johnson , 73–87. Ashgate.

[cars70016-bib-0083] Staggenborg, S. , and H. Ramos . 2016. “The LGBT Movement.” In Social Movements, edited by S. Staggenborg , 132–154. Oxford University Press.

[cars70016-bib-0084] Statistics Canada . 2017a. “Toronto [Census Metropolitan Area], Ontario and Canada [Country] (Table). Census Profile, Ethnic Origin. 2016 Census.” Statistics Canada Catalogue. Accessed November 29, 2017, https://www12.statcan.gc.ca/census-recensement/2016/dp-pd/prof/details/page.cfm?Lang=E&Geo1=CMACA&Code1=535&Geo2=PR&Code2=01&SearchText=toronto&SearchType=Begins&SearchPR=01&B1=Ethnic%20origin&TABID=1&type=0.

[cars70016-bib-0085] Statistics Canada . 2017b. “Toronto [Census Metropolitan Area], Ontario and Canada [Country] (Table). Census Profile, Visible Minority Population. 2016 Census.” Statistics Canada Catalogue. Accessed November 29, 2017, https://www12.statcan.gc.ca/census-recensement/2016/dp-pd/prof/details/page.cfm?Lang=E&Geo1=CMACA&Code1=535&Geo2=PR&Code2=01&SearchText=toronto&SearchType=Begins&SearchPR=01&B1=Visible%20minority&TABID=1&type=0.

[cars70016-bib-0086] Suppes, A. , J. Van Der Toorn , and C. T. Begeny . 2021. “Unhealthy Closets, Discriminatory Dwellings: The Mental Health Benefits and Costs of Being Open About One's Sexual Minority Status.” Social Science & Medicine 285: 114286. 34365070 10.1016/j.socscimed.2021.114286

[cars70016-bib-0087] Svensson, J. , and C. Strand . 2023. “The Promise of Double Living. Understanding Young People With Same‐Sex Desires in Contemporary Kampala.” Journal of Homosexuality 71, no. 8: 201–2029. 10.1080/00918369.2023.2218958 37262126

[cars70016-bib-0088] Timmermans, S. , and I. Tavory . 2012. “Theory Construction in Qualitative Research: From Grounded Theory to Abductive Analysis.” Sociological Theory 30, no. 3: 167–186.

[cars70016-bib-0089] Vaccaro, A. , and R. M. Koob . 2019. “A Critical and Intersectional Model of LGBTQ Microaggressions: Toward a More Comprehensive Understanding.” Journal of Homosexuality 66, no. 10: 1317–1344. 30403566 10.1080/00918369.2018.1539583

[cars70016-bib-0090] Williams, C. L. , P. A. Giuffre , and K. Dellinger . 2009. “The Gay‐Friendly Closet.” Sexuality Research and Social Policy 6, no. 1: 29–45.

[cars70016-bib-0091] Yip, A. K. T. 2004. “Negotiating Space With Family and Kin in Identity Construction: The Narratives of British Non‐Heterosexual Muslims.” Sociological Review 52, no. 3: 336–350.

[cars70016-bib-0092] Zaidi, A. U. , A. Couture‐Carron , E. Maticka‐Tyndale , and M. Arif . 2014. “Ethnic Identity, Religion, and Gender: An Exploration of Intersecting Identities Creating Diverse Perceptions and Experiences With Intimate Cross‐Gender Relationships Amongst South Asian Youth in Canada.” Canadian Ethnic Studies 46, no. 2: 27–54.

